# Labour's Health Policy

**Published:** 1923-02

**Authors:** 


### LABOUR'S HEALTH POLICY.

THE pamphlet on "The Labour Movement and Preventive and Curative Medical Services." which has been published by the Trades Union Congress and the Labour Party, is a sufficient indication, if one were needed, that the Labour Party will press for an extensive programme of health legislation. The sweeping character of the policy of the party with regard to health, which this pamphlet is declared to be, can only be appreciated by a study of the document itself. Though poverty and bad industrial and housing conditions are agreed to be mainly responsible for the serious amount of ill-health which is constantly being revealed by statistics, medical training and the present methods of medical practice tend, says the document, to aggravate the evil.

### A Public Medical Service.

The remedy proposed is a complete public medical service, the aim of which must be "to put every discovery of modern medical science, every class of specialist, every comfort of the best type of nursing home, sanatorium, or hospital within the reach of all—rich and poor." The consultants and specialists should be as far as possible whole-time salaried officers, and this would apply to the general practitioners and to "a sufficient number of dentists, midwives, nurses and dispensers." More than double the present accommodation of the voluntary hospitals, which are to disappear, would be necessary, with a complete scheme of treatment centres everywhere.

### The Cost!

With the proposals for the complete co-ordination of all existing health services under one common control, there will be agreement in many quarters. When, however, we come to the vast comprehensive scheme of publicly-provided medical services, to be available for everyone in the land, the economic factor is too airily treated in a single paragraph in the pamphlet, which says that "the cost of the health services should be a charge upon communal funds," and that the improved health of the workers "would soon mean an increased production of wealth, more than counterbalancing the whole cost of the health services." This sort of vague generalisation too often occurs with regard to the vital economic questions underlying all these sweeping programmes of reform, and detracts seriously from the value of the pamphlet as a real contribution to the solution of the problems which await the attention of the Minister of Health and his advisers.

---

In the Holophane Filterlite, the Holophane Co. has made a new development in the control of light. The Filterlite is a two-piece bowl having plain exterior surfaces with an interior prismatic construction designed so to control the light rays as to give a wide light distribution with the maximum of efficiency and the minimum of contrasts and glare. The Filterlite is both useful and ornamental as the lower diffusing zone has a permanent enamelled inner surface with an ornamental exterior design, and, unlike units of the open bowl and semi-enclosing type, the Filterlite does not rapidly become full of dust. It may be specially recommended for any building where general even illumination is required without sharp contrasts due to the brightness of the source of light and to harsh shadows.

